# Olivocochlear Changes Associated With Aging Predominantly Affect the Medial Olivocochlear System

**DOI:** 10.3389/fnins.2021.704805

**Published:** 2021-09-03

**Authors:** Sergio Vicencio-Jimenez, Madison M. Weinberg, Giuliana Bucci-Mansilla, Amanda M. Lauer

**Affiliations:** ^1^The Center for Hearing and Balance, Department of Otolaryngology-Head and Neck Surgery, Johns Hopkins University School of Medicine, Baltimore, MD, United States; ^2^Laboratorio de Neurosistemas, Departamento de Neurociencia, Facultad de Medicina, Universidad de Chile, Santiago, Chile

**Keywords:** superior olivary complex, auditory efferents, olivocochlear system, age related hearing loss, aging

## Abstract

Age-related hearing loss (ARHL) is a public health problem that has been associated with negative health outcomes ranging from increased frailty to an elevated risk of developing dementia. Significant gaps remain in our knowledge of the underlying central neural mechanisms, especially those related to the efferent auditory pathways. Thus, the aim of this study was to quantify and compare age-related alterations in the cholinergic olivocochlear efferent auditory neurons. We assessed, in young-adult and aged CBA mice, the number of cholinergic olivocochlear neurons, auditory brainstem response (ABR) thresholds in silence and in presence of background noise, and the expression of excitatory and inhibitory proteins in the ventral nucleus of the trapezoid body (VNTB) and in the lateral superior olive (LSO). In association with aging, we found a significant decrease in the number of medial olivocochlear (MOC) cholinergic neurons together with changes in the ratio of excitatory and inhibitory proteins in the VNTB. Furthermore, in old mice we identified a correlation between the number of MOC neurons and ABR thresholds in the presence of background noise. In contrast, the alterations observed in the lateral olivocochlear (LOC) system were less significant. The decrease in the number of LOC cells associated with aging was 2.7-fold lower than in MOC and in the absence of changes in the expression of excitatory and inhibitory proteins in the LSO. These differences suggest that aging alters the medial and lateral olivocochlear efferent pathways in a differential manner and that the changes observed may account for some of the symptoms seen in ARHL.

## Introduction

As social animals, any circumstance that disrupts our ability to communicate can have profound consequences on our health. Age-related hearing loss (ARHL) is the most common sensory impairment among the elderly and is the third leading health condition overall in older adults (Collins, 1997). ARHL is defined as a progressive loss of hearing ability that is most pronounced at high frequencies (Bowl and Dawson, 2019). Notably, it is also characterized by the deterioration of sound localization ability and a reduction in speech recognition, especially in noisy environments (Dubno et al., 1984; Stuart and Phillips, 1996; Frisina and Frisina, 1997; Gordon-Salant, 2005; Shojaei et al., 2016). About half of people over 70 years of age have hearing impairments severe enough to reduce their communication abilities (Agrawal et al., 2008; Yamasoba et al., 2013), which is associated with social isolation, depression, accelerated cognitive decline, and increased risk of dementia (Lin, 2011; Livingston et al., 2017; Loughrey et al., 2018; Rutherford et al., 2018). Despite its prevalence and the negative health outcomes associated with ARHL, our understanding of the biological mechanisms and processes that explain this condition is still incomplete, particularly with respect to our knowledge of how the central auditory pathways are altered and the role they play in aging. These gaps in our knowledge of ARHL are even more prominent with respect to the efferent auditory pathways, which are not usually the focus of aging research.

In mammals, the auditory efferent pathways form a network composed of feedback loops that includes the auditory cortex and subcortical nuclei such as the thalamus, inferior colliculus, superior olivary complex and cochlear nucleus (Malmierca and Ryugo, 2011). Although recent results have presented evidence of direct projections to the cochlea from the ventral nucleus of the lateral lemniscus (Suthakar and Ryugo, 2021), virtually all efferent projections from the central nervous system to the cochlea leave from the superior olive. Thus, the final component of this efferent system that reaches the cochlea is known as the olivocochlear (OC) system, which originates in the superior olivary complex (SOC) (Rasmussen, 1946). The OC system is comprised of two neuronal groups: (i) the medial olivocochlear neurons (MOC) and (ii) the lateral olivocochlear neurons (LOC) (Warr and Guinan, 1979). Large MOC neurons are located in the medial periolivary region, predominantly in the ventral nucleus of the trapezoid body (VNTB) in rodents and send myelinated projections that make axo-somatic synapses with the outer hair cells of the cochlea (OHC) (Guinan et al., 1983; Brown, 2011; Fuchs and Lauer, 2018). Synapses are organized tonotopically, with greater density in the middle regions of the cochlea in most species (Guinan, 1996; Maison et al., 2003). LOC neurons are smaller than their MOC counterparts and originate in the lateral superior olive (LSO) (Guinan et al., 1983; Brown, 2011). They send unmyelinated fibers to the cochlea that make axo-axonal synapses with auditory nerve type I fibers near the inner hair cells (Guinan, 1996; Simmons, 2002). They also display a tonotopic organization, with slightly more innervation in the apical half of the cochlea (Guinan et al., 1984; Robertson et al., 1987; Liberman et al., 1990). Both MOC and LOC neuron populations release acetylcholine as their main synaptic transmitter (Bobbin and Konishi, 1971; Vetter et al., 1991; Eybalin, 1993; Blanchet et al., 1996). LOC neurons express a greater diversity of neurotransmitters than MOCs, including dopamine (DA), calcitonin gene-related peptide (CGRP), GABA, and opioid peptides such as enkephalin (Eybalin, 1993; Ciuman, 2010; Reijntjes and Pyott, 2016; Wu et al., 2020).

Activation of the MOC pathway produces a reduction in cochlear sensitivity (Mountain, 1980; Murugasu and Russell, 1996; Cooper and Guinan, 2006). Functionally, the MOC pathway has been shown to facilitate stimulus discrimination in noise, enhance auditory selective attention, and protect against damage from noise exposure (Winslow and Sachs, 1987; Kawase and Liberman, 1993; May et al., 2004; Terreros et al., 2016; Lauer et al., 2021). Less is known about the function of the LOC system, with current evidence being limited and sometimes contradictory. However, evidence suggests that LOC neurons can modulate the activity of type I auditory nerve fibers (Felix and Ehrenberger, 1992; Groff and Liberman, 2003; Wu et al., 2020). The efferent activity of the LOC potentially protects IHC and nerve fibers from acoustic overexposure (Wu et al., 2020) or could be modulating the set point of auditory nerve fibers, thus contributing to the generation a range of spontaneous firing rates (Le Prell et al., 2003; Ciuman, 2010; Wu et al., 2020).

Most of the available evidence for the role of OC pathways in ARHL comes from physiological assessments of the OC system and studies of inner ear structures. Rodent models have shown that age-related changes in the MOC system plays a role in the progression of ARHL (Jacobson et al., 2003; Zettel et al., 2007; Zhu et al., 2007; Boero et al., 2020). Also, experimental lesions of the olivocochlear bundle suggest that efferent feedback contributes to slowing cochlear aging (Liberman et al., 2014). In addition to this, there is evidence that OC pathways are weakened in aging humans (Parthasarathy, 2001; Kim et al., 2002). Furthermore, changes in cochlear efferent innervation have been observed in aging mice and humans (Lauer et al., 2012; Zachary and Fuchs, 2015; Liberman and Liberman, 2019; Jeng et al., 2020; Kobrina et al., 2020).

There is remarkably little work focused on alterations at the central level of the aging olivocochlear system (Radtke-Schuller et al., 2015). In the present work, we studied age-associated changes in the brainstem regions of the olivocochlear auditory efferent system of mice. We quantified the number of cholinergic OC neurons at different ages and compared them with auditory brainstem response (ABR) measurements in quiet and noise conditions. In addition, markers of excitatory [vesicular glutamate transporter 1 (VGLUT1)] and inhibitory [glutamic acid decarboxylase 65-kilodalton isoform (GAD65)] synapses were also assessed. These results were compared with age-related cell loss in the vestibular efferent and trigeminal motor nucleus to determine if age-related degeneration is specific to the auditory efferents.

## Materials and Methods

### Animals

The experiments were performed using a total of 65 adult CBA/CaJ mice (30 males and 35 females). The original breeding pairs were obtained from The Jackson Laboratory (strain #000654). Mice were bred and housed in a quiet, low-traffic vivarium at Johns Hopkins University (Wu et al., 2020). The mice were housed in groups with *ad libitum* water and food and under a 12–12-h night/day cycle. This strain was selected because CBA/CaJ mice show a hearing loss and cochlear damage trajectory across the lifespan that is similar to humans (Sergeyenko et al., 2013; Kobrina and Dent, 2020; Kobrina et al., 2020). The subjects ranged from 1 to 30 months old. Mice in the range of 1–8 months (*n* = 33; 18 males and 15 females) were considered young adults and those in the range of 18–30 months were considered old (*n* = 32; 12 males and 20 females). All procedures were approved by the Johns Hopkins University Institutional Animal Care and Use Committee and followed NIH guidelines for the care and use of laboratory animals.

### Auditory Brainstem Response (ABR)

To evaluate hearing status, ABRs were recorded in quiet and noise backgrounds. Mice were anesthetized with an i.p. injection of 100 mg/kg ketamine and 20 mg/kg xylazine. To prevent corneal damage, an ophthalmic ointment was applied to the eyes. Once anesthetized, mice were placed inside a sound-attenuating chamber 10 cm from a speaker (MF1, TDT), measured from the pinnae. Mice were placed on a heating pad to maintain a temperature of 37°C ± 1°C. Subdermal platinum needle electrodes were placed at the vertex of the skull (+electrode), over the left bulla (−electrode) and in the muscle of the ipsilateral hind leg (ground electrode).

The ABR signal recorded by the electrodes was sent to a head stage (Medusa4Z, TDT) and then transferred to a pre-amplifier (RA4PA, TDT) with 20-fold amplification. The signals were acquired with a sampling rate of 12 kHz and bandpass filtered (highpass 0.3 kHz and lowpass 5 kHz), notched at 50 Hz, and averaged over 512 presentations. Off-line and prior to analysis, the signal was filtered again at 0.3–3 KHz.

The SigGenRZ software (TDT) was used to program the stimulus protocols for click and tones and generated using the TDT BioSigRZ platform. The stimuli consisted of clicks (0.1 ms square wave pulse of alternating polarity) and 5 ms tone pulses of 8, 12, 16, and 24 kHz (0.5 ms onset/offset), played at a rate of 21 repetitions/s. We decided not to evaluate the 32 KHz frequency because, in our experience, it was unlikely to find an ABR response at this frequency in old animals (Kobrina et al., 2020). All stimuli were presented at descending levels in 10 dB increments, starting at 90 to 0 dB. Stimuli were calibrated using a 1/4″ free-field microphone (Bruel & Kjaer) placed at the location where the mouse head would normally be during testing, using the BioSigRZ software (TDT). For the experiment in which the ABR stimuli were masked with background noise, a second speaker (MF1, TDT) was used, which was positioned 15 cm from the animal’s right ear and at 90° with respect to the stimulus presentation (ABR) speaker. Through this speaker, and during the entire presentation of the ABR stimuli, a broadband noise of 40 dB SPL was presented. This intensity was measured at the level of the right pinna of the mouse.

Testing lasted between 70 and 90 min. After the recording was finished mice were placed in an individual cage over a heating pad and monitored until recovered. Once fully awake, they were returned to their home cages.

Auditory brainstem response recordings were analyzed offline using BioSigRZ and MATLAB (vR2019a; MathWorks). The amplitudes and latencies of waves 1 to 3 (click responses at 90 dB SPL) were measured offline manually by two independent examiners blinded to the animals’ condition. Wave 4 and 5 were not reliably detected in the recording with background noise, so we did not quantify them. The ABR threshold was calculated automatically, using a custom MATLAB script. The threshold was defined as the sound intensity level at which the peak-to-trough amplitude of the ABR wave was at least two standard deviations above the mean baseline amplitude estimated from the last 5 ms in the recording when no sound stimulus was present (Lina and Lauer, 2013; McGuire et al., 2015; Lauer, 2017; Schrode et al., 2018). A value of 95 dB SPL was used as a threshold for cases where one could not be found.

### Brainstem Sectioning

Mice were deeply anesthetized with sodium pentobarbital (i.p., 150 mg/kg) followed by an intracardiac injection of heparin (0.7 ml/kg). Immediately, the mice were transcardially perfused with 60 ml of a 4% paraformaldehyde solution. The brain tissue was removed and postfixed in 4% paraformaldehyde at 4°C for a minimum of three and up to 18 h. To perform the brain sections, the frontal portion of the cerebrum was removed (1 mm rostral from the confluence of the sinuses) allowing the regions of interest (ROI) to sit in their coronal plane. This remaining portion of the brain was embedded in a solution containing 5 ml of albumin gel mixed with 0.4 ml of 5% glutaraldehyde and 1 ml of 37% paraformaldehyde. The brain was mounted on a LeicaVT1200S vibratome and sectioned in the coronal plane in 50 μm slices. Serial sections through the auditory brainstem were placed in Tris-NaCl buffer (TBS).

### Immunostaining

The brain sections were first incubated in a permeabilizing solution (TBS with 0.5% Triton X-100) for 15 min and blocking buffer (TBS with 10% normal rabbit serum) for 1 hr. The sections were incubated with the primary antibody for choline acetyltransferase (CHAT) diluted in TBS (1:400; Millipore AB144P) and 0.5% of Triton X-100 for 20 h at room temperature on a shaker. Afterward, the brain sections were incubated with secondary antibodies diluted in TBS (1:200 Biotinylated Rabbit Anti-Goat; Vector Labs BA-5000), for 1 h at room temperature on a shaker. Then, they were incubated with ABC reagent for 1 h at room temperature (ABC kit PK-6100) on a shaker. Finally, sections were treated for 5 ± 1 min with a solution containing 0.05% DAB, 0.4% nickel ammonium sulfate, and 0.01% hydrogen peroxide. In order to dilute and wash the reagents, the sections were rinsed with TBS three times between each of the steps described above.

In the case of the GAD65 and the VGLUT1 antibodies the procedures were similar, except for the primary (anti-GAD65 1:1000; Abcam ab26113 and anti-VGLUT1 1:1000; Thermo Fisher Scientific 48-2400) and secondary antibodies (1:200 Biotinylated goat anti-mouse; Vector Labs BA-9200 and 1:200 Biotinylated goat anti-rabbit; Vector Labs BA-1000 for GAD65 and VGLUT1, respectively). For more details, see Schrode et al. (2018).

To have negative controls, in each brain we omitted the primary antibody in one section and the secondary antibody in an additional section. Finally, the stained sections were mounted on glass slides, air-dried, mounted with Permount (Fisher Scientific), and coverslipped.

### Cell and Area Quantification in Regions of Interest

Sections including the LSO, VNTB, vestibular efferent nuclei, and trigeminal motor nuclei were photographed at 40× on a microscope (Labophot; Nikon) with a mounted CCD camera (Progres; Jenoptik). To include both the LSO and VNTB in the same image, overlapping photographs were taken and merged using Fiji (Rueden et al., 2017). This procedure was also performed to combine the images of the trigeminal nuclei. The ROI were identified manually using a graphics tablet and stylus (Cintiq 22HD; Wacom). An automatic threshold algorithm was used in Fiji to identify the immunoreactive areas for CHAT, VGLUT1, and GAD65 antibodies. To determine the best suitable algorithm, all available automatic threshold algorithms in Fiji were evaluated against manual results from a blind observer in a reduced data set. It was determined that of the algorithms tested, Default, RenjiEntropy and Triangle algorithm was the best at identifying immunoreactive zones for CHAT, VGLUT1, and GAD65, respectively. To correct for illumination or staining intensity irregularities, we applied a leveling adjustment to each section, so that the mean pixel intensity within each ROI was equivalent across all sections of each animal (Schrode et al., 2018). To identify positive immunolabeling, we made a histogram of pixel intensity based on all pixels within the ROIs across all slices (Schrode et al., 2018). Label density was quantified as the fraction between the total number of thresholded pixels and the total number of pixels within each ROI.

In each of the sections, the number of positive CHAT cells per region of interest was determined using the Fiji cell counting plugin. The cell numbers shown correspond to the values of each ROI from one hemisphere. Cell counting was done by an observer who was blind to the age and hearing status of the animals. To reduce the possibility of double counting, cell count estimates were corrected using the Abercrombie method (Abercrombie, 1946). A second independent observer counted a fraction of the samples (25% of the total). No significant differences were found between the two observers.

### Statistical Analysis

For our comparisons between young and old mice we determined that animals between 2 and 8 months of age qualified as part of the young adult group, while those older than 18 months qualified as old mice. This criterion was based on previous aging brackets determined in mice (Flurkey et al., 2007).

Statistical analyses were performed with MATLAB or GraphPad prism. Statistical test information and sample numbers are specified in the Figure Legends and in the Results. The error bars correspond to the standard error of the mean (SEM). For comparisons between two different groups (such as the number of cells between young and old mice) we used an unpaired *t* test with Welch’s correction. For cases in which we evaluated how a response (such as ABR threshold) was affected by two factors (age and presence of background noise) we used a two-way ANOVA or a mixed effect analysis, both with a Sidak’s multiple comparisons test. Statistical significance was defined as: *p* > 0.05 not significant (n.s.), *p* < 0.05 (^∗^), *p* < 0.001 (^∗∗^), and *p* < 0.0001 (^∗∗∗^).

## Results

### Olivocochlear Cell Count

**FIGURE 1:**
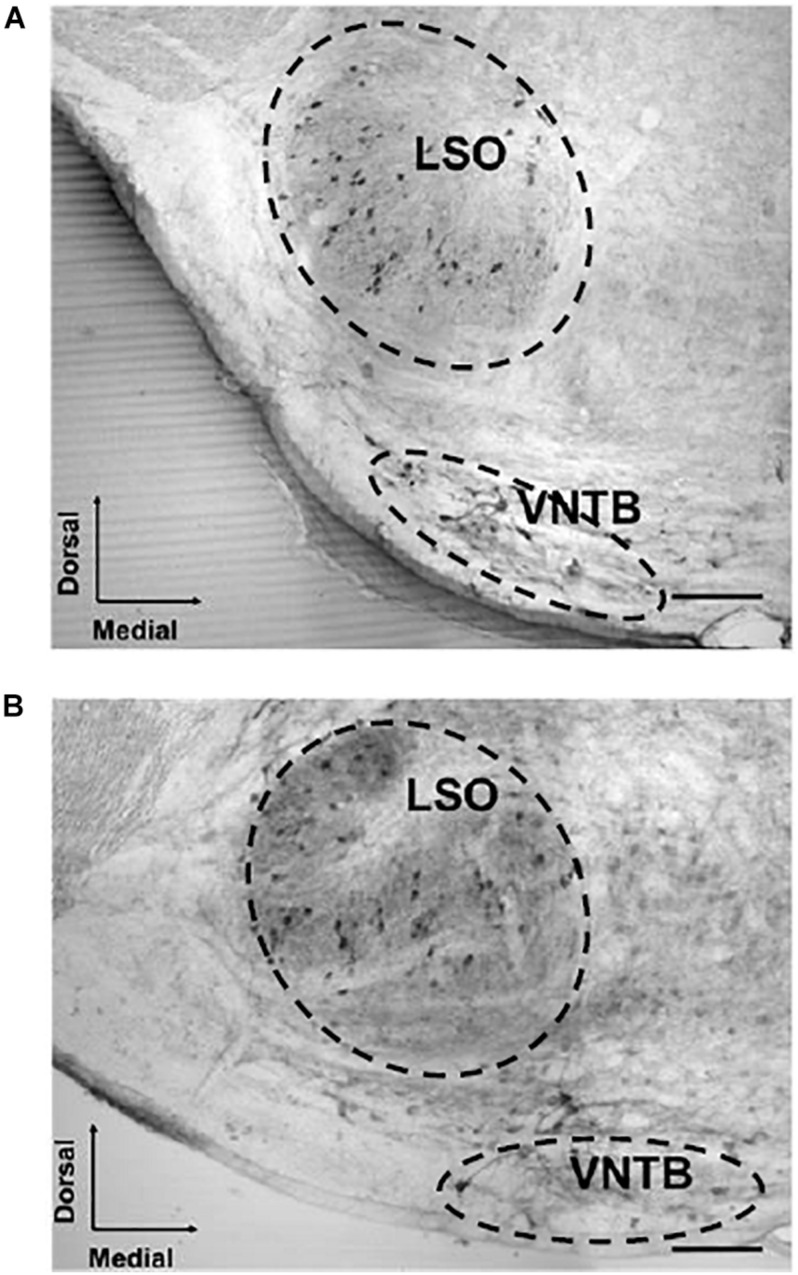
Examples of acetylcholinesterase-stained sections of the SOC of a young and aged mouse. The image shows two photomicrographs (20×) of a cross section through the left side of the mouse brainstem. The image in panel **(A)** corresponds to a 2-month-old male mouse and panel **(B)** to a 26-month-old male mouse section. The somas of MOC neurons are located in the VNTB and LOC neurons can be seen in the LSO. 100 μm scale bar. LOC, lateral olivocochlear system; LSO, lateral superior olive; MOC, medial olivocochlear system; SOC, superior olivary complex; VNTB, ventral nucleus of the trapezoid body.

**FIGURE 2:**
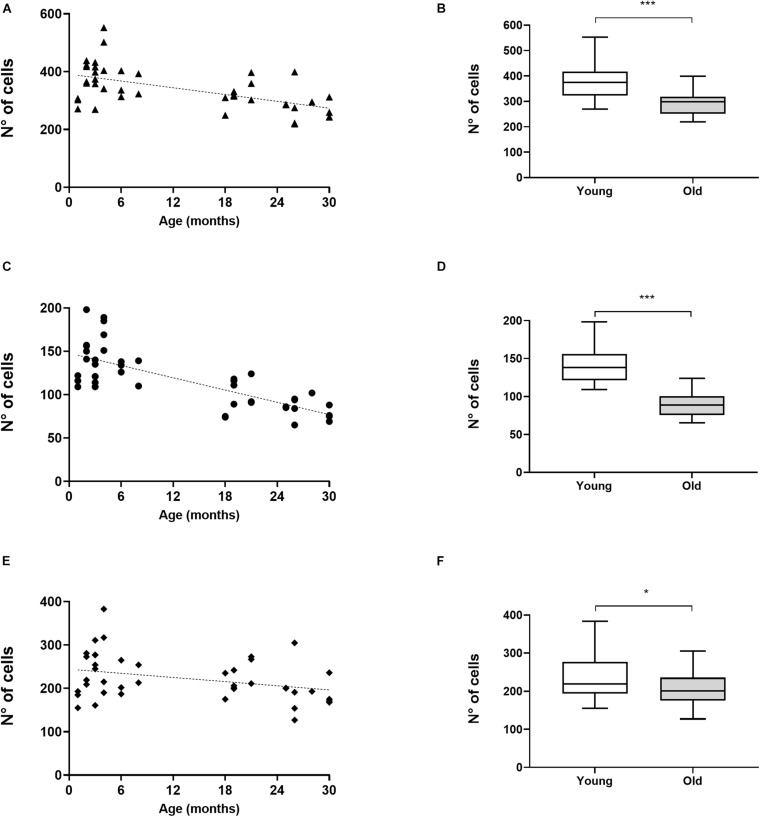
Aging-associated decrease in number of OC cells predominantly affects MOC cells. The image shows the comparisons in the number of CHAT-immunoreactive cells observed in the SOC of young (*n* = 23) and aged (*n* = 20) animals. The figures in the left column show the number of cells for each of the animals, where each black symbol represents an individual animal and the dotted line the fitted line. The total number of OC cells is shown in panel **(A)**, while in panel **(C)** is the total number of MOC cells counted in the VNTB and in panel **(E)** the number of LOC cells in the LSO is shown. Linear regressions in panels **(A,C,E)** showed statistical significance (**A:** R-square = 0.3344, *F* = 20.60, DFn = 1, DFd = 41, *p* < 0.0001; **C:** R-square = 0.5809, *F* = 56.84, DFn = 1, DFd = 41, *p* < 0.0001; **E:** R-square = 0.1101, *F* = 5.074, DFn = 1, DFd = 41, *p* < 0.0297). The figures in the right column show boxplots comparing the numbers of CHAT-positive cells between young and aged animals. Panel **(B)** shows the comparison for all OC cells, **(D)** for MOC and **(F)** LOC cells. A significant decrease in cell numbers was found in aged animals for OC cells **(B)** MOC **(D)** and LOC cells **(F)** (Unpaired *t* test with Welch’s correction, **B:** *p* < 0.0001, *t* = 4.433, df = 39.96; **D:** *p* < 0.0001, *t* = 7.611, df = 37.99; **F:** *p* = 0.0409, *t* = 2.112, df = 40.59). CHAT, choline acetyltransferase; LOC, lateral olivocochlear system; LSO, lateral superior olive; MOC, Medial olivocochlear system; OC, olivocochlear system; SOC, superior olivary complex; VNTB, ventral nucleus of the trapezoid body. The *p*-values are defined as: *p < 0.05 and ***p < 0.0001.

We assessed the number of CHAT-immunoreactive cells in the SOC in 23 young mice (1–8 months of age) and in 20 old mice (18–30 months of age). In the SOC we identified two large populations of neurons that had dark staining, one located in the VNTB and the other in the LSO (Figure 1). In the VNTB we found larger cells that we classified as neurons of the MOC system, whereas those found in the LSO were classified as part of the LOC system (Brown and Levine, 2008; Radtke-Schuller et al., 2015). The number of total OC cells and their respective MOC and LOC subdivisions for the different ages are shown in Figure 2. We evaluated potential differences in cell number between male and female mice. We found no significant differences between sexes, so we pooled together the male and female data. The number of cells counted in young animals were within the range previously described in mice (Brown and Levine, 2008), 378.3 ± 69.10 for total OC cells, 139.6 ± 26.26 for MOC and 238.1 ± 54.92 for LOC. When comparing young and old animals, significant decreases in the total number of OC cells were observed in the aged animals (Figures 2A,B). We found a significant correlation between the age of the animals and the number of OC neurons (Figure 2A, R-square = 0.3344, *F* = 20.60, DFn = 1, DFd = 41, *p* < 0.0001), with aging associated with decreased number of cells. When comparing the young and old mouse groups, we found a 21% significant decrease in the average number of CHAT-positive neurons in the aged mice (Figure 2B, *p* < 0.0001, *t* = 4.433, df = 39.96). When performing these comparisons between young and old animals for the two subdivisions of the OC system, we found that both cell populations showed significant age-associated decreases (Figures 2C–F). However, the magnitude of this reduction was not uniform. There was a significant correlation between increasing age and a decrease in number of MOC cells (Figure 2C, R-square = 0.5809, *F* = 56.84, DFn = 1, DFd = 41, *p* < 0.0001), with a 36% average decrease in old mice (Figure 2D, *p* < 0.0001, *t* = 7.611, df = 37.99). The reduction in the number of LOC cells as a function of age was less pronounced (Figure 2E, R-square = 0.1101, *F* = 5.074, DFn = 1, DFd = 41, *p* < 0.0297), with an average reduction of 13% in the number of LOC cells in the old mice (Figure 2F, *p* = 0.0409, *t* = 2.112, df = 40.59). Thus, these results indicate that the reduction of total number of OC cells during normal aging is mainly driven by a decrease in the number of MOC neurons.

**FIGURE 3:**
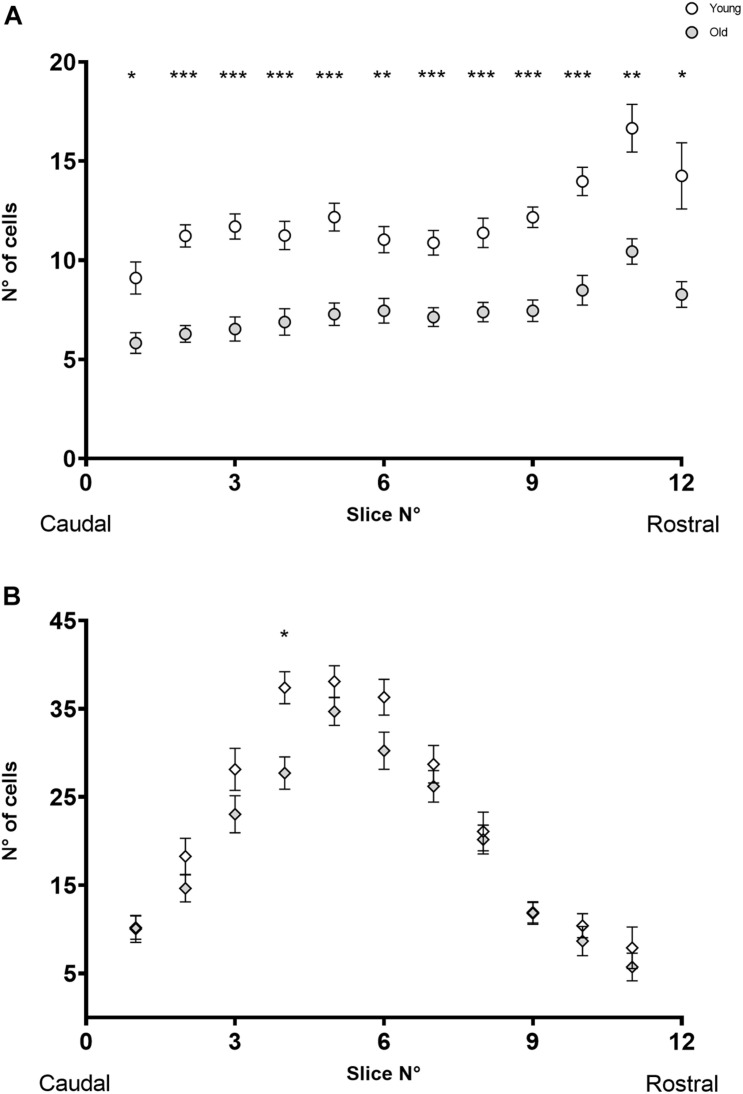
The image shows the number of CHAT-immunoreactive cells found as a function of rostro-caudal localization in the SOC in young (*n* = 23) and old (*n* = 20) animals. In panel **(A)** is pictured the distribution of MOC cells, where the empty circles represent the young mice, and the gray circles represent the old animals. The image in panel **(B)** shows the distribution for the LOC cells, where the empty rhombus represents the young mice, and the gray rhombus represent the old ones. A mixed effect analysis found significant differences in the distribution of MOC cells of young vs. old mice (*p* = < 0.0001, *F* (1, 41) = 116.0) and the subsequent Sidak multiple comparisons test found significant differences in all the slides. In the case of LOC cells, a mixed effect analysis found significant differences in the distribution of MOC cells of young vs. old mice (*p* = < 0.0456, *F* (1, 41) = 4.251) and a Sidak multiple comparisons test found significant differences in slide number 4 (*p* = 0.0063). Individual points show the mean value, and the error bars indicate the standard error of the mean. Each slide had a width of 50 μm. CHAT, choline acetyltransferase; LOC, lateral olivocochlear system; MOC, medial olivocochlear system; SOC, superior olivary complex. The *p*-values are defined as: *p < 0.05, **p < 0.001 and ***p < 0.0001.

To investigate whether cell losses were restricted to specific regions along the rostro-caudal axis, or if there were group differences that could not be appreciated only by considering the total number of cells per specimen, we compared the distribution of MOC and LOC cells along the rostro-caudal axis in young and old animals. Figure 3 shows the number of MOC (Figure 3A) and LOC (Figure 3B) cells as a function of the rostro-caudal axis. For both neuronal populations we found no significant age-associated changes in the pattern of cell distribution in the rostro-caudal axis (Mixed-effects analysis with no significant interaction between age and distribution). In the case of MOC cells, a Sidak’s multiple comparisons test between young and old mice yielded significant differences in all observed slides (Figure 3A, with *p* values between 0.04 and <0.0001), indicating that there was a uniform loss of neurons from old animals along the entire axis. In contrast, significant differences were only found in one section of the rostro-caudal LOC axes (Figure 3B, slide number 4, Sidak’s multiple comparisons test *p* = 0.0063). These results reinforce the observation that age-related OC neuronal loss is dominated by MOC cells.

### Auditory Brainstem Response

**FIGURE 4:**
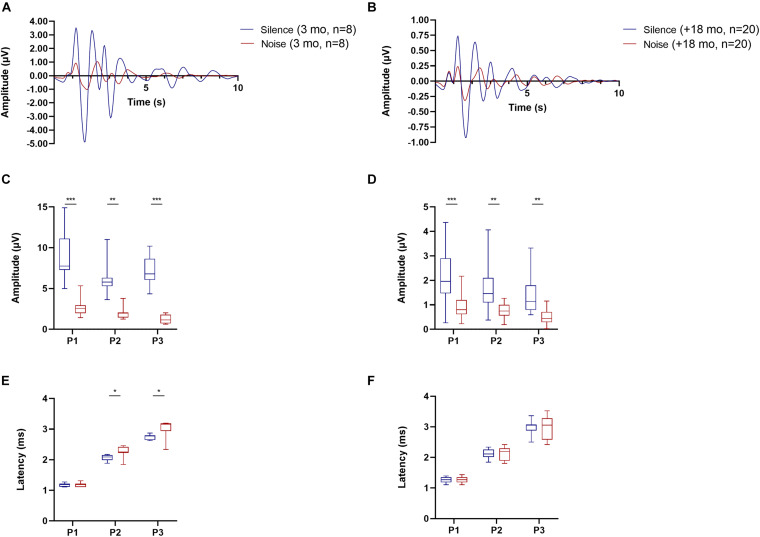
Auditory brainstem response (ABR) waves for young (3 months, *n* = 8) and old mice between (18–30 months, *n* = 20) under conditions of silence and background noise. The average ABR waveforms recorded at a 90 dB SPL click for young mice is shown in panel **(A)** and in panel **(B)** for old mice. Blue lines represent the mean values in μV under quiet conditions and the red lines the mean in the presence of 40 dB SPL background noise. Panels **(C,D)** shows box and whiskers plots representing the amplitude for ABR waves 1–3 for young **(C)** and old **(D)** mice. The box and whiskers graph in panels **(E,F)** represent the latency for the peak of ABR waves 1–3 for young and old mice, respectively. Blue boxes show the results in quiet conditions and the red ones under noise (40 dB SPL). Asterisks signal the presence of significant differences between quiet and noise (two-way ANOVA, with a Sidak’s multiple comparisons test). The *p*-values are defined as: *p < 0.05, **p < 0.001 and ***p < 0.0001.

**FIGURE 5:**
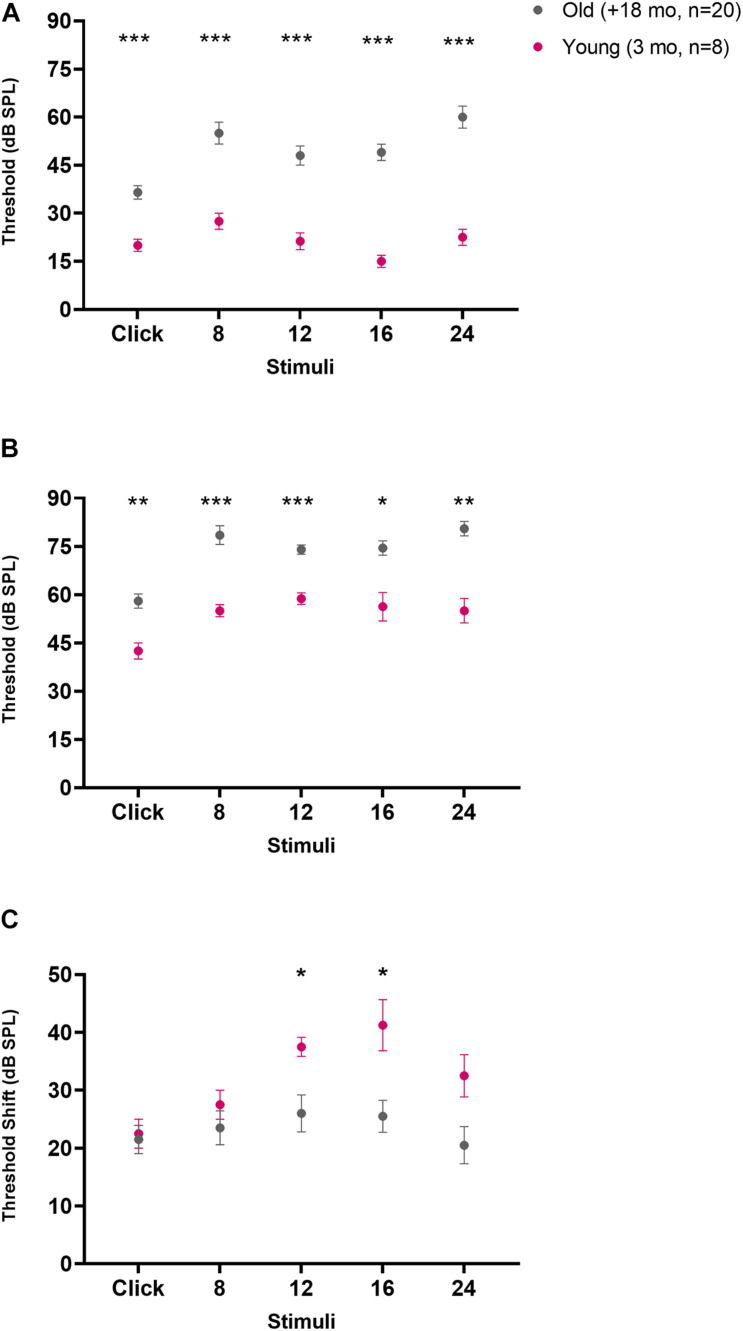
Auditory brainstem response (ABR) thresholds for young (3 months, *n* = 8) and old mice between (18–30 months, *n* = 20) under conditions of silence and background noise. Panels **(A,B)** shows the ABR thresholds for young and old mice in quiet and noise conditions, respectively. In panel **(C)** the threshold shift between quiet and noise for young and old mice is shown. The pink circles represent the average threshold value for young animals and the gray circles the average threshold value for the old mice. The error bars show the standard error of the mean. Asterisks signal the presence of significant differences between the young and old animal groups (two-way ANOVA, with a Sidak’s multiple comparisons test). The *p*-values are defined as: *p < 0.05, **p < 0.001 and ***p < 0.0001.

In addition to quantifying age-related changes in OC cell number, we also evaluated the auditory responses of young (*n* = 8, 3 months) and old animals (*n* = 20, 18–30 months) with ABRs in quiet conditions and in the presence of background noise (BBN of 40 dB SPL, Figures 4, 5). The ABR waveforms (Figures 4A,B) and thresholds measured in our animals tested in silence (Figure 5A) were consistent with what is expected for CBA/CaJ mice of those ages (Kobrina et al., 2020). Regarding the wave amplitudes, we found significant reduction in the peak to trough amplitude of the ABR waves 1–3 in presence of a masking noise compared to quiet background, both for young (Figure 4C; two-way ANOVA *p* < 0.0001, *F* (1, 42) = 98.91; Sidak’s multiple comparisons *p* < 0.0001) and old mice (Figure 4D bottom; two-way ANOVA *p* < 0.0001, *F* (1, 114) = 60.84; Sidak’s multiple comparisons *p* < 0.0001). In terms of the ABR wave peak latencies, in young animals we found an increase in the latencies for waves 2 and 3 in the presence of noise compared to quiet (Figure 4E bottom; two-way ANOVA *p* = 0.0013, *F* (1, 42) = 11.94; Sidak’s multiple comparisons *p* = 0.0443 for wave 2 and *p* = 0.0039 for wave 3). In the case of the old animals, we found no significant differences in wave 1–3 latencies between the quiet and noise conditions.

Auditory brainstem response thresholds for the young animals ranged between 20.0 ± 5.3 dB SPL for clicks to 27.5 ± 7.1 dB SPL for 8 kHz stimuli (Figure 5A pink circles). The threshold values for the old animals were distributed in a range from 36.5 ± 9.3 dB SPL for click stimuli to 60.0 ± 15.4 dB SPL for 24 kHz stimuli (Figure 5A gray circles). We found significant differences between young and old animals in thresholds in silence for all stimuli (Figure 5A; two-way ANOVA *p* < 0.0001, *F* (1, 26) = 49.49; Sidak’s multiple comparisons test with all values of *p* < 0.0001). When considering ABRs in the noise conditions (Figure 5B) we observed that in young mice thresholds ranged between 42.5 ± 7.1 dB SPL for clicks to 58.75 ± 5.2 dB SPL for 12 kHz stimuli (Figure 5B pink circles). In addition, when compared to the silent condition, this increase in threshold was significant (two-way ANOVA *p* < 0.0001, *F* (1, 14) = 125.1; Sidak’s multiple comparisons test with all values of *p* < 0.0001). In the case of the old mice, the noise thresholds ranged from 58.0 ± 9.8 dB SPL for click stimuli to 80.5 ± 10.0 dB SPL for 24 kHz stimuli (Figure 5B gray circles). This increase in threshold was also statistically significant when compared to the silent condition (two-way ANOVA *p* < 0.0001, *F* (1, 38) = 67.95; Sidak’s multiple comparisons test with all values of *p* < 0.0001). Furthermore, when contrasting the thresholds of young and old animals in the presence of noise, we found that for all stimuli, old animals presented significantly higher thresholds than young animals (Figure 5B; two-way ANOVA *p* < 0.0001, *F* (1, 26) = 51.99; Sidak’s multiple comparisons with values between *p* = 0.0181 and *p* < 0.0001). It is interesting to highlight that the magnitude of the change in thresholds between quiet and noise was not the same for young and old animals (Figure 5C). In the presence of noise, the ABR thresholds for young animals had an average increase of 32.25 ± 7.5 dB SPL, whereas in old animals the average increase was 23.40 ± 2.4 dB SPL. We found significant differences between the silence-to-noise threshold shift between the young and old mice group, observing a greater shift in threshold for young animals at the 12 and 16 KHz stimuli (Figure 5C; two-way ANOVA *p* = 0.0291, *F* (1, 26) = 5.337; Sidak’s multiple comparisons test *p* = 0.0181 for 12 KHz and *p* = 0.0481 for 16 KHz).

**FIGURE 6:**
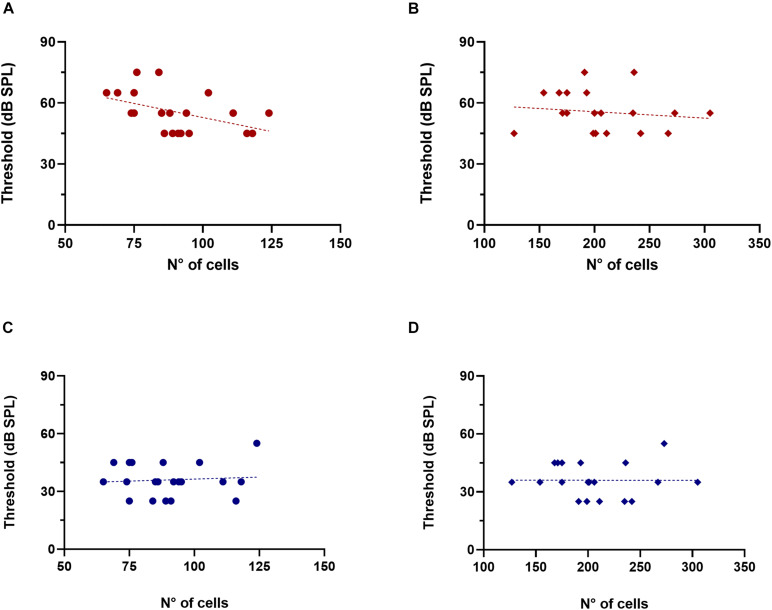
Association between click ABR thresholds in noise and the number of MOC cells in old animals. The figure shows the values of click ABR thresholds in the presence of noise (red) and in silence (blue) as a function of the number of MOC (**A,C**, circles) and LOC cells (**B,D**, rhombuses). The dotted lines correspond to the fitting curve. A significant correlation was found between the number of MOC cells and ABR thresholds in the presence of background noise [**(A)** R-square = 0.2138, *F* = 4.896, DFn = 1, DFd = 18, *p* = 0.0401]. LOC, lateral olivocochlear system; MOC, medial olivocochlear system.

Next, we evaluated whether there were correlations between ABR thresholds in quiet and noise and the number of OC neurons in old mice (Figure 6). Considering that the MOC system is involved in hearing in noise, and that one of the main symptoms of ARHL is an impairment of this ability, we were particularly interested in observing whether there was a correlation between ABR thresholds in noise and the number of MOC cells. Notably, we found that animals with higher numbers of MOC neurons tended to present lower click ABR thresholds in noise conditions (Figure 6A, R-square = 0.2128, *F* = 4.896, DFn = 1, DFd = 18, *p* = 0.0401). In contrast, we found no significant trends with the number of MOC cells for ABRs in silence, nor in any condition analyzed as a function of LOC cell number (Figures 6C,D). We also performed these same analyses in young mice (*n* = 8, 3 months), where we found no correlation between ABR threshold values (in quiet and noise) and the number of olivocochlear cells (MOC and LOC).

**FIGURE 7:**
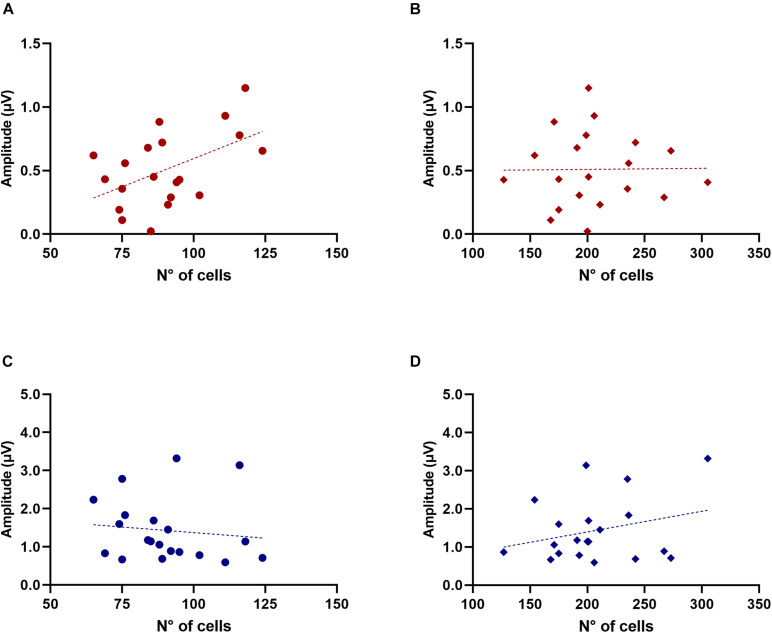
Association between Wave 3 amplitude in noise and the number of MOC cells in old animals. The figure shows ABR wave 3 amplitude in the presence of noise (red) and in silence (blue) as a function of the number of MOC (**A,C**, circles) and LOC cells (**B,D**, rhombuses). The dotted lines correspond to the fitting curve. A significant correlation was found between the number of MOC cells and wave 3 amplitude in the presence of background noise [**(A)** R-square = 0.2577, *F* = 6.248, DFn = 1, DFd = 18, *p* = 0.0223]. LOC, lateral olivocochlear system; MOC, medial olivocochlear system.

In addition to this, we also evaluated the potential associations between ABR wave amplitude (wave 1–3) in quiet and noise conditions with the number of OC neurons. For the old animal group, waves 1 and 2 showed no correlations with MOC cells. But, for wave 3, we found that mice with higher numbers of MOC cells also had higher ABR wave 3 amplitudes in background noise conditions (Figure 7A R-square = 0.2577, *F* = 6.248, DFn = 1, DFd = 18, *p* = 0.0223) but not in quiet (Figure 7C). In contrast, we found no significant correlation for the case of LOC cells (Figures 7B,D). For the case of young animals (*n* = 8, 3 months) we found no correlations in any condition.

### Alterations in Synaptic Markers

**FIGURE 8:**
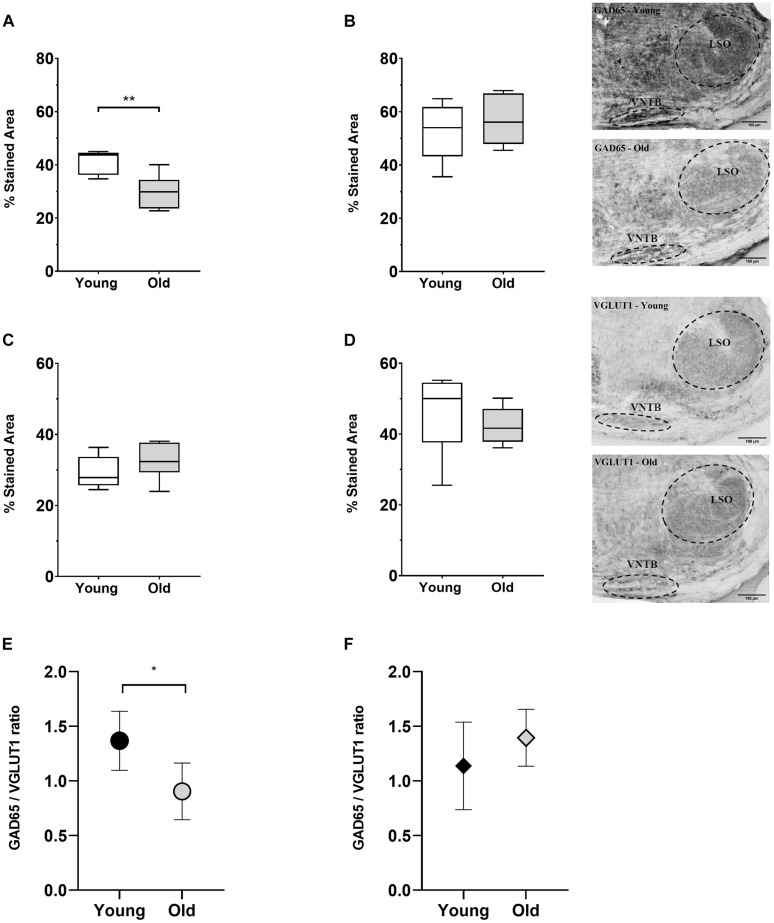
Age-associated changes of inhibitory and excitatory synaptic markers. The first row shows boxplots representing the fraction of GAD65-immunoreactive area of young (*n* = 5) and old mice (*n* = 6, gray-filled boxes) in the VNTB **(A)** and LSO **(B)**. For the VNTB in **(A)**, a significant difference was found between young and old mice (unpaired t test with Welch’s correction, *p* = 0.0081, *t* = 3.401, df = 8.850). The images on the right correspond to an example of GAD65 staining at 10x in a young male mouse (top, 3 m.o.) and an old male mouse (bottom, 26 m.o.). The second row is showing boxplots that represent the fraction of VGLUT1-immunoreactive area of young (*n* = 5) and old mice (*n* = 6, gray-filled boxes) in the VNTB **(C)** and LSO **(D)**. On the right, there are two examples of staining of VGLUT1 at 10x for a young female mouse (top, 2 m.o.) and an old female mouse (bottom, 28 m.o.). The last row shows a comparison of the GAD65/VGLUT1 ratio between young and old animals, where **(E)** represents the VNTB and **(F)** the LSO, and the error bars indicate the standard error of the mean. In the case of VNTB **(E)**, a significant difference was found between the two groups (unpaired t test with Welch’s correction, *p* = 0.0192, *t* = 2.881, df = 8.507). GAD65, glutamic acid decarboxylase 65-kilodalton isoform; LSO, lateral superior olive; VGLUT1, vesicular glutamate transporter 1; VNTB, ventral nucleus of the trapezoid body. The *p*-values are defined as: *p < 0.05 and **p < 0.001.

Complementary to the previous observations, we studied potential age-associated changes in the VNTB and LSO of two synaptic markers: (i) one GABAergic (GAD65) and (ii) one glutamatergic (VGLUT1) (Figure 8). We decided to use GAD65 because, under our previous experience, this antibody strongly labels synaptic terminals (Schrode et al., 2018). We quantified the fraction of the area in our ROI that was stained with VGLUT1 or GAD65 in 10 young animals (2–3 months) and 12 old animals (18–28 months). It is important to clarify that this was a separate group from the CHAT-processed animals and, furthermore, that the immunolabeling was not performed on the same mice (five young and six old were used for each condition). These specimens were part of our laboratory archives, obtained from mice used in ABR and cochlear anatomy experiments reported in Kobrina et al. (2020). The first column of Figure 8 shows the results obtained for VNTB (Figures 8A,C,E) while the second column shows the values for LSO (Figures 8B,D,F). Within the VNTB we found a significant age-associated decrease in the labeled area for GAD65 (Figure 8A, *p* = 0.0081, *t* = 3.401, df = 8.850). We did not find significant changes in the labeled area for GAD65 in the LSO (Figure 8B), nor for any of the regions with VGLUT1 (Figures 8C,D).

To estimate age-associated changes in the relative amount of excitatory to inhibitory markers in our ROI, we calculated the ratio between the GAD65- and VGLUT1-labeled area for young and adult animals (Figures 8E,F). In the case of the VNTB we found a significant decrease in the GAD65/VGLUT1 ratio in old mice (Figure 8E, *p* = 0.0192, *t* = 2.881, df = 8.507). In the case of the LSO, we did not find any significant differences. This evidence suggests that during aging, in addition to a loss in neuronal number, the VNTB and the MOC system undergo alterations in the balance of inhibitory and excitatory signals.

### Comparison With Other Brainstem Efferent Nuclei

**FIGURE 9:**
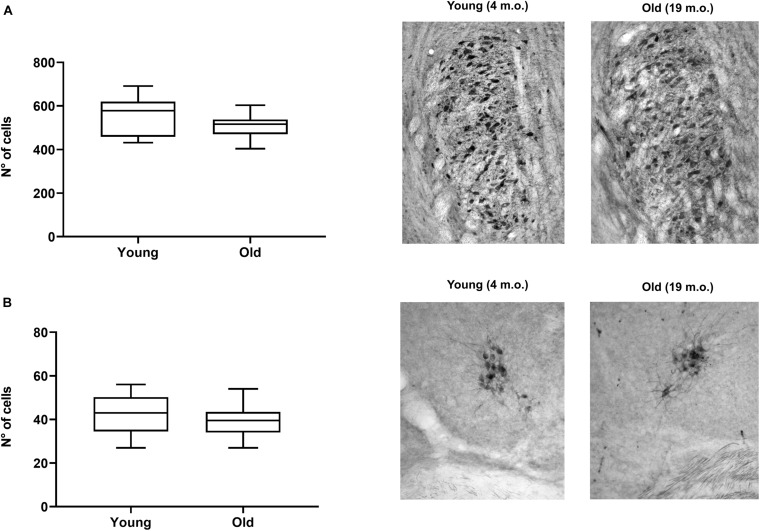
Comparison of trigeminal and vestibular efferent cell numbers in young (*n* = 10) and aged mice (*n* = 10). The figure shows boxplots comparing the number of CHAT-positive cells between young and aged animals for the trigeminal motor nucleus **(A)** and the vestibular efferent nucleus **(B)**. The images on the right of each boxplot correspond to photomicrographs examples of trigeminal **(A)** and vestibular efferent **(B)** sections stained with acetylcholinesterase for young and old mice. We found no significant differences between young and old mice in neither of the two cases. CHAT, choline acetyltransferase.

To evaluate whether the observed changes were specific to OC neurons or reflect degeneration of cholinergic neurons in brainstem more generally, we quantified the differences in the number of CHAT-immunoreactive cells in young (*n* = 10) and old (*n* = 10) mice in two other brainstem efferent regions: (i) the trigeminal motor nucleus and (ii) the vestibular efferent nucleus (Figures 9A,B, respectively). Although both regions showed slight decreases in the average number of stained cells in old animals (8% for the trigeminal and 7% for the vestibular efferent), we did not find significant differences between the number of cells in young and old animals in either region. This again reinforces the idea that the MOC system could be showing a distinct and significantly greater reduction in cell numbers than other brainstem efferent regions.

## Discussion

In this study, we quantified the loss of mouse olivocochlear neurons as a function of age, coupled with ABR measurements in silence and noise. Additionally, we quantified age-associated changes in one GABAergic and one glutamatergic synaptic marker in the LSO and VNTB. We found significant age-associated alterations specific to the MOC system, including (i) a 36% decrease in the number of CHAT-labeled MOC neurons, (ii) a correlation between MOC cell number and ABR threshold in noise, and (iii) a decrease in the GAD65/VGLUT1 ratio in the VNTB. In contrast, the changes we found in LOC were of a smaller extent. We found a lesser decrease in the number of LOC cells (12%) which was not associated with changes in the ABR response. In addition, we also did not find significant changes in the number of CHAT-labeled cells in the vestibular efferent and trigeminal motor nucleus neurons. These observations indicate differential vulnerability to aging of MOC cells and provide evidence for their role in ARHL.

The results we obtained regarding the number of OC, trigeminal, and vestibular cells in young animals were within the expected ranges for mice (Sturrock, 1987; Campbell and Henson, 1988; Brown and Levine, 2008; Mathews et al., 2015). In old animals, the loss of OC neurons was dominated by MOC loss (Figures 2D, 3A) being on average 2.7 times greater than the loss of LOC cells. This decline in neuronal number was symmetrical between both hemispheres and along the rostro-caudal axis, with little inter-subject variability and no significant differences between male and female mice. Such differential reduction led to changes in the ratio of LOC to MOC cells in the OC system, from an average of 1.7:1 LOC per MOC in young mice to 2.3:1 in old mice.

These findings are congruent with previous evidence observed in studies that have explored age-related OC alterations in mice and humans. Age-associated decreases in contralateral suppression of distortion product otoacoustic emission levels have been observed in humans and CBA mice (Jacobson et al., 2003). Considering that this response is mediated by the MOC system, even if middle ear muscles contribute to the effect (Xu et al., 2017; Valero et al., 2018), this is consistent with age-related MOC dysfunction. Moreover, anatomical research in mice and humans also supports these differential changes. There is a loss in the density of cochlear MOC neurons as a function of age, while the density of LOC innervation does not decrease overall (Liberman and Liberman, 2019; Jeng et al., 2020; Kobrina et al., 2020). However, other age-associated changes in LOC synapses have been identified. There is a synaptic rearrangement involving an increase in efferent innervation of IHCs in mice with accelerated ARHL, in which the aging cochlea recovers some features of postnatal development with the re-emergence of efferent inhibition of the IHCs (Lauer et al., 2012; Zachary and Fuchs, 2015). All this evidence points to a loss of MOC neurons and synapses with the OHC, accompanied by changes in LOC synaptic organization without substantial loss of neuronal cell bodies.

Moreover, the absence of observed changes in the number of CHAT-labeled cells in the trigeminal motor nucleus, vestibular efferents, and LOC also is in line with the changes expected in the aging brain, where we would not anticipate seeing large reductions overall in the number of neurons, except in specific regions (Morrison and Hof, 1997; Pannese, 2011). There are interesting similarities between our results and findings from research conducted in aging gerbils that also quantified the number of OC, trigeminal and vestibular efferent cell bodies (Radtke-Schuller et al., 2015). This study also found that the decrease in cell number was restricted to the OC system, with no alterations in other efferent pathways. This suggests a greater vulnerability to aging of the OC system compared to other efferent regions of the brainstem. However, for gerbils the OC decrease was distributed in both MOC and LOC subpopulations, without changes in the ratio of MOC and LOC cells in old animals. This species difference in the vulnerability to OC neurons could be related to the particular susceptibility of gerbils to auditory brainstem degeneration from exposure to environmental noise over the course of a lifetime (Statler et al., 1990; McGinn and Faddis, 1994, 1998; Gleich and Strutz, 2002).

The mechanisms underlying the vulnerability of the OC system and, especially the MOC, cannot be directly concluded from our results, but there are at least three factors that could be contributing: (i) differential alterations in the brainstem regions, (ii) a specific vulnerability of MOC cells, or (iii) differential alterations in peripheral synapses.

For the first factor, our data describing the age-dependent changes in the fraction of GAD65 and VGLUT1 suggest that VNTB shows alterations that are not observed in LSO (Figure 8). The decrease in the fraction of GAD65 and consequently the GAD65/VGLUT1 ratio could be reflecting a decline in the proportion of inhibitory inputs in the region. These results are also in line with previous publications reporting an age-related decrease in GABA and GAD levels in other central regions of the auditory system (Caspary et al., 1990; Burianova et al., 2009; Ibrahim and Llano, 2019). In addition to the functional effects that may result from the decrease in GABA-mediated inhibition (Caspary et al., 2008), there is also the possibility that it may be contributing to the vulnerability of VNTB, potentially through excitotoxicity effects.

On the other hand, the absence of observed changes in the LSO is consistent with previous results in gerbils, where no age-related effects on GABAergic and glycinergic markers were found (Gleich et al., 2004). However, our data has several caveats that limit our interpretation. The GAD65 and VGLUT1 measurements did not come from the same animals, although the animals were raised and aged in the same environment during the same time periods, and the specimens were prepared by the same experimenter. Also, we did not capture all the excitatory and inhibitory inputs, for instance those that are positive for VGLUT2 instead of VGLUT1. It is also unclear if the GAD65 only represents GABAergic or also GAD-positive glycinergic inputs. Future experiments will tease apart the contributions of different sources of inputs to OC neurons to the observed age-related effects.

Regarding the specific vulnerability of MOC neurons, changes in somatic expression levels of the Kv3.1 channel have been observed in MOC cells of aged mice (Zettel et al., 2007), suggesting a higher vulnerability than their LOC counterparts. Complementary to this, extracellular recordings from the LSO in rats have shown that neuronal populations do not significantly change their responses with age, supporting the idea of their resistance to degeneration (Finlayson and Caspary, 1993). Another notable difference between MOC, LOC and vestibular efferent cells is the presence of myelin sheath in MOCs. While intuition suggests that myelin should be a protective factor in the face of trauma and aging (Ceballos et al., 1999; Reeves et al., 2005), there are situations where it has been observed to be more vulnerable (Fujimura et al., 1991; Tang et al., 1997). It is also possible that the loss of MOC neurons is related to other factors such as a higher energy demand, which the sole presence of myelin is not necessarily a good predictor (Harris and Attwell, 2012).

There is evidence supporting the idea that changes at the level of the cochlea may be contributing to the observed differences in loss of LOC and MOC neuron cell bodies. For example, the modifications in the number of MOC efferent neurons could be explained by degeneration and decrease in the number of OHCs, whereas IHCs remain much more intact with age (Jeng et al., 2020; Kobrina et al., 2020; Wu et al., 2020). In addition, aged OHCs manifest morphological alterations, such as a reduction in size, that precede the decrease in the number of MOC efferent fibers (Jeng et al., 2020). In contrast, IHC numbers do not decrease with age to the same extent as OHCs do with age (Spongr et al., 1997; Sergeyenko et al., 2013; Kobrina et al., 2020). Rather, there is a subpopulation of IHCs that reverts into an immature-like biophysical and morphological profile (Corns et al., 2018), which are possibly be the ones that are re-innervated by inhibitory cholinergic efferents (Lauer et al., 2012; Corns et al., 2018).

Regarding our ABR results, the thresholds we identified in young and old animals were within the expected ranges, as were the wave 1–3 latencies and amplitudes (Henry, 1979; Schrode et al., 2018; Kobrina et al., 2020). In this context, the observed decrease in ABR amplitude in old mice compared to young mice indicates the existence of a reduced physiological response to sound. The absolute increase in the ABR threshold in quiet condition is also a reflection of this (Figure 5). When looking at our results from shifts in thresholds between the quiet and noise it is noticeable that older animals had smaller shifts than the young adults. This result is consistent with previous findings in mice and may be reflecting OHC damage or ceiling effects due to the already increased thresholds (Ehret, 1979). However, inferring the exact cause of these findings would be extremely challenging. Previous evidence shows that the changes associated with ARHL in these mice are multifactorial, which makes it difficult to attribute a single main cause of ARHL to a particular type of cell degeneration. For example, Kobrina et al. (2020) showed that multiple alterations at the cochlear level, including loss of hair cells, synaptic ribbons, and changes in the stria vascularis are related to ARHL.

One of the most remarkable findings was the correlation between the number of MOC cells in old animals with the ABR thresholds (Figure 6A) and with the wave 3 amplitudes (Figure 7A) in noise conditions. These correlations were not found in young animals. There is previous evidence showing that the MOC system can suppress the cochlear response to continuous noise and, thereby, it can help unmask transient tone stimuli, making them more detectable (Winslow and Sachs, 1988; Liberman and Guinan, 1998). Therefore, it is reasonable to have found that a higher number of MOC cells correlated with lower ABR thresholds in noise. It is also worthy of note that the superior olive is directly related with wave 3 (Buchwald and Huang, 1975; Henry, 1979; Melcher et al., 1996) and, that P3 and N3 are lost with the application of local anesthetic into the trapezoid body (Wada and Starr, 1983). Given that this manipulation results in non-specific effects in the trapezoidal body, the precise contribution of MOC neurons is unknown. However, it does show that diminished function in the vicinity of the MOC cell bodies reduces this ABR component, which supports the idea that this region is altered in aging. On the other hand, it is also reasonable that no correlations were observed with waves 1 and 2 of the ABR. The amplitude of wave 1 is dominated by age-related cochlear degeneration that is well documented in this strain, including in our laboratory (Kobrina et al., 2020). In addition, age-related increases in olivocochlear innervation of inner hair cells may further reduce the auditory nerve activity that generates ABR wave 1 (Lauer et al., 2012; Zachary and Fuchs, 2015; Kobrina et al., 2020). Therefore, it is difficult to infer how the loss of MOC inputs would independently contribute to the already diminished wave 1. Centrally generated ABR waves often show non-linear changes in response to peripheral damage and diminished ABR wave 1 (for instance, as shown by our lab in Schrode et al., 2018).

The absence of this correlation with wave 3 in young animals was also reasonably expected. This is due to the fact that the ABR thresholds and the number of neurons were distributed across narrower ranges in young animals. In an aged and impaired system, it is more probable that the decrease in the number of MOC cells has an observable impact on signal processing in noise since redundant mechanisms for hearing in noise may be diminished by age-related cochlear and central degeneration. Other functions such as protection against acoustic trauma and selective attention could also be affected by the decrease in MOC numbers and may contribute to further deterioration of auditory pathway and cognitive functions. Further experiments are needed in order to explore these questions.

## Data Availability Statement

The raw data supporting the conclusions of this article will be made available by the authors, without undue reservation.

## Ethics Statement

The animal study was reviewed and approved by Johns Hopkins University Institutional Animal Care and Use Committee.

## Author Contributions

AL: original idea, project administration and supervision, and funding acquisition. AL and SV-J: experimental conceptualization and data analysis. SV-J: experimental development and manuscript writing. SV-J and MW: tissue processing. SV-J and GB-M: image processing. AL, SV-J, and GB-M: manuscript editing. All authors contributed to the article and approved the submitted version.

## Conflict of Interest

The authors declare that the research was conducted in the absence of any commercial or financial relationships that could be construed as a potential conflict of interest.

## Publisher’s Note

All claims expressed in this article are solely those of the authors and do not necessarily represent those of their affiliated organizations, or those of the publisher, the editors and the reviewers. Any product that may be evaluated in this article, or claim that may be made by its manufacturer, is not guaranteed or endorsed by the publisher.
